# Oral-health-related background factors and dental service utilisation among Sudanese children with and without a congenital heart defects

**DOI:** 10.1186/s12903-016-0318-5

**Published:** 2016-11-15

**Authors:** H. M. Ali, M. Mustafa, E. F. Nasir, S. A. Lie, S. Hasabalrasol, O. H. Elshazali, R. W. Ali, M. S. Skeie

**Affiliations:** 1 Department of Clinical Dentistry, Faculty of Medicine and Dentistry, University of Bergen, Bergen, Norway; 2 Oral Health Centre of Expertise in Western Norway, Hordaland, Bergen, Norway; 3 Department of Community Dentistry, University of Science and Technology, Khartoum, Sudan; 4 Ahmed Gasim hospital, Khartoum Teaching Hospital, Khartoum, Sudan; 5 Ahmed Gasim hospital, Faculty of Medicine University of Khartoum, Khartoum, Sudan; 6 Department of Periodontics, University of Science and Technology, Khartoum, Sudan

**Keywords:** Caries, Children, Congenital heart defects, Dental health services, Gingivitis, Oral health background factors

## Abstract

**Background:**

Sudanese children with congenital heart defects (CHDs) were found to have poorer oral health than those without CHDs. The aims of this study were to: describe the patterns of oral-health-related background factors in children with and without CHD and explore any differences, and to evaluate the effects of background factors on caries and gingivitis prevalence and dental services utilisation.

**Methods:**

In this analytical cross-sectional study, caregivers of children aged 3–12 years with (CHD cases *n* = 111) and without CHDs (Controls *n* = 182), underwent face-to-face interviews using a structured questionnaire. The questionnaire items covered several oral health background factors (independent variables) including: child’s health status, oral hygiene practices, dental services utilization, mother’s level of education, and caregiver’s perception and awareness of their child’s oral health. The relationship between these factors and occurrence of ‘*caries*’ and ‘*gingivitis*’ as well as *‘child’s dental services utilisation’* (dependent variables) were explored using multiple adjusted and hierarchal logistic regression analyses.

**Results:**

Compared with controls, CHD cases had lower frequencies of brushing and use of fluoridated toothpaste, and their caregivers were less knowledgeable about caries. Among CHD cases, the variables (brushing and fluoridated toothpaste use) had significant impacts on caries prevalence (odd ratio (OR) =5.6, 95% confidence interval (CI): 1.4–22.8 and OR = 0.3, 95% CI: 0.1–0.8 for infrequent compared to frequent ones, respectively) as well as the mother’s level of education (OR = 2.6, 95% CI: 1.0–6.4). When differences in background factors were controlled for, the adjusted ORs for caries and gingivitis prevalence in CHD cases compared with controls were 1.8, (95% CI: 1.1–3.2) and 5.3 (95% CI: 2.9–9.4), respectively. Among CHD cases, the child’s age (8–12 years: OR = 11.9, 95% CI: 1.9–71.6), and the mother’s level of education (lower education: OR = 0.2, 95% CI: 0.03–0.9) were significantly associated with the child’s dental services utilisation.

**Conclusions:**

Lower frequencies of brushing and use of fluoride tooth paste were reported among CHD cases, and brushing had the predominant significant impact on caries prevalence. The child’s age and the mother’s level of education were the main factors affecting the child’s (CHD cases) dental services utilisation.

**Electronic supplementary material:**

The online version of this article (doi:10.1186/s12903-016-0318-5) contains supplementary material, which is available to authorized users.

## Background

Congenital heart defects (CHDs) and structural abnormalities of the heart affect ~8 in 1000 live births worldwide [1]. Generally, caries and periodontal diseases have low mortality, however, both have high morbidity and are responsible for dental pain, teeth loss as well as being risk factors for other systemic complications especially among children [2]. Children with a CHD are at a disadvantage in that, because of their underlying medical condition, development of an oral disease can put their general health at risk [3]. The main risks and conditions caused by odontogenic bacteraemia are life-threatining conditions, including infective endocarditis and brain abcesses [4]. Recent changes in the recommendations for the prevention of infective endocarditis in children with a CHD have limited antibiotic prophylaxis to certain types of CHD and highlighted the importance of non-antibiotic prophylaxis [5, 6]. It has been emphasised that finding ways to prevent oral health problems in children with a CHD is more important than ever [5–7]. The role of the caregiver of a child with a CHD, in terms of practising daily oral health activities and possessing oral health knowledge, has been recognised to be more important than previously thought [6, 8–10].

Previous studies on the dental knowledge and attitudes of children with a CHD and their caregivers have yielded unsatisfactory results with regard to oral hygiene practices and the use of dental services [3, 11–13]. A study conducted in Brazil in 2002 found that 28.8% of children with a CHD had never visited a dentist [10]. The authors also reported that 47.1% of these children brushed their teeth more than once daily, which was below expectations despite the fact that brushing was performed with parental supervision [10]. A study undertaken in the UK in 1996 showed that caregivers of children with a CHD attended fewer regular dental check-ups for their children than caregivers of children with no medical problems [14]. In studies from several developing countries, caregivers of children with a CHD reported with less awareness about the link between CHDs and oral diseases and the increased risk of their children developing infective endocarditis [9, 10, 15]. However, in studies from other developed countries, caregivers had fairly better knowledge [16, 17].

Findings vary regarding the susceptibility to dental diseases of children with compared to children without a CHD resident in industrialised countries [14, 16, 18, 19]. In developing countries, few epidemiological studies have been conducted on children with a CHD and little information is available about their oral health status [9, 20, 21]. However, in a recent study in Khartoum state, Sudan, we found a significantly higher prevalence of caries and gingivitis among children aged 3–12 with a CHD years than among those without a CHD [22]. As a developing country, Sudan is markedly different from industrialised countries, not only in terms of cultural and socio-economic backgrounds, but also in terms of the dental care system [23, 24]. In Sudan, there is a lack of available and accessible regular dental care [25] and the provision of preventive dental care is not prioritised [25]. Thus, oral-health-related background factors may be distributed differently in Sudan compared with developed countries.

As in other countries, oral-health-related background factors can differ markedly among subgroups within the population [26, 27]. As stated earlier, our previous study in Khartoum state, Sudan, children with a CHD were found to exhibit poorer oral health than those without a CHD [22]. To gain a more in-depth understanding of this finding, detailed mapping of the underlying risks and oral health factors among that sample of children is required. Such mapping should consider oral hygiene practices, the caregiver’s oral-health-related knowledge, and the availability and utilisation of dental services. Additional aspects of interest are the caregivers’ perceptions of their children’s oral health and how satisfied they are with it. Such a study will reveal inadequacies related to oral health background factors, and will enhance efforts towards provision of better oral health care for all children, especially among those with a CHD. Therefore, in this study, we aimed to: 1) describe the patterns of oral health background factors in children with and without a CHD and explore any differences; 2) evaluate the effects of background factors on the prevalence of caries and gingivitis among these children; and 3) evaluate the influence of background factors on their utilisation of dental services.

## Methods

### Ethical approval

For patients with a CHD, ethical approval was obtained from Ahmed Gasim Hospital (Sudan), Sudan Federal Ministry of Health, Research Ethical Committee at the University of Science and Technology (Sudan), and from the Regional Committee for Medical Research Ethics Western Norway (No. 2265). For the controls, ethical approval was obtained from the Sudan Ministry of Education (Khartoum), and Sudan Ministry of Primary and Pre-school Education in the three Khartoum localities (Khartoum city, Khartoum North and Omdurman). Ethical permission was obtained from the local offices of the Ministry of Education in each of the selected localities and permission letters were taken to each of the selected schools. Participants received verbal and written information about the study and confidentiality was ensured. They were also given the option of withdrawing from the study at any time without giving any reason. A translated consent form was then completed and signed by the participants’ guardians upon agreement to participate (both CHD cases and controls).

### Study design, setting and population

#### Study design

This was an analytical cross-sectional study among a group of children with CHDs (CHD cases), compared with children without CHDs (controls).

#### Sample size

The sample size was calculated initially to estimate the number of children needed to detect differences in caries between children with and without CHD. This was done using the sample size equation of two-sided Student’s *t*-test (the test to be used for comparisons). The smallest difference to be detected in the mean (dmft/DMFT: decayed, missing and filled teeth) between the two groups was 1 and the variance was estimated to be 2.0 in the controls and 2.5 in the CHD cases. The level of significance was set at 0.05 and the power at 80%. The estimated sample size was 60 CHD cases and 60 controls in each age group: age group 1 (3–7 years) with deciduous teeth; and age group 2 (8–12 years) with permanent incisors and first molars. Altogether, the estimated sample consisted of 240 participants in the CHD case and control groups. Caregivers were invited to participate and answer the questionnaire while accompanying their children.

#### Sampling technique

One hundred and seventeen CHD cases and their caregivers from the Ahmed Gasim Cardiac Center in Khartoum, Sudan were invited to participate and 111 of them consented to participation (purposive sampling). Caregivers were included when they had a child with a confirmed diagnosis of CHD between the age of 3 and 12 years. The control group comprised children without CHD along with their caregivers; about 190 were invited and 182 consented to participate from schools and kindergartens in Khartoum State. A stratified random sampling technique was used to select controls through age and gender group matching to the CHD cases and the strata were rural–urban. Around 60 controls from each of the three localities of Khartoum State (Khartoum city, Khartoum North and Omdurman) were enrolled. All participants were approached once and since the response rates in both groups were sufficient no further attempts were undertaken.

#### Study population

Participants were the caregivers of children with a CHD (111 CHD cases, 62 girls) attending routine cardiac check-ups at the Ahmed Gasim Cardiac Centre, and the caregivers of 182 children (89 girls) without a CHD (controls) attending schools and kindergartens in the same state. The caregivers of children with and without a CHD were divided into age group 1 (caregivers of children aged 3–7 years [primary teeth]) and age group 2 (caregivers of children aged 8–12 years [permanent teeth]). The variable *‘child’s age’* was dichotomized to age 1 (0) if age was 3–7 years and age 2 (1) if age was 8–12 years*.* The variable *‘child’s gender’* was dichotomized to boy (0) or girl (1). More information on calibration and reliability tests for the clinical examinations is provided in our previous study, of which this study is a continuation [22].

#### Data collection tools

The data were collected through individual face-to-face interviews with the children’s caregivers. Interviews were conducted by two trained research assistants, including the principle investigator, who initially approached and invited participants after they had been informed about the study by a cardiologist. Most of the items were adopted from a questionnaire used in 2009 to assess the oral health status of 12-year-old Sudanese children and was previously tested for validity, and reliability and recommended the use of face-to-face interviews [28]. However, the final structured and closed-ended questionnaire underwent some modifications and included some extra items to assess the caregivers’ knowledge about dental caries. This resulted in a questionnaire consisting of 25 questions, which were directly translated from English into Arabic during the interview. Pilot testing of the questionnaire was not feasible because CHD cases were not easily available to complete the desired sample size within the study period. Therefore, CHD cases that met the inclusion criteria were included without having a pilot group. A similar questionnaire design was used earlier and the testing revealed no major flaws. Face-to-face interviews were preferred over a self-administered questionnaire because the presence of illiterate caregivers was anticipated, and this was recommended after the pilot testing of a similar questionnaire in the earlier mentioned study.

### Independent variables

#### Status

The dichotomous variable *‘status’* was categorised into the presence (‘CHD case’; 1) or absence (‘control’; 0) of a CHD.

#### Mother’s education

The variable *‘mother’s education’* consisted of five items ranging from illiterate, primary schooling, secondary schooling to university or higher education. A significant difference in the number of illiterate mothers between CHD cases and controls (19.4 vs 3%) was identified in our previous study on the same sample [22]. The education variable was dichotomised into: low educational level (primary education or less) and high educational level (secondary education to university education) [29] and was included as a background variable in the present regression models.

#### Caregiver’s perceptions

The two items that measured the caregiver’s perceptions concerned the child’s oral health and the appearance of the child’s teeth. Two variables were constructed from the previous items; the variable *‘perception of oral health’* was categorised into ‘good oral health’ (0) when the caregiver perceived that their child had ‘good’ or ‘very good’ oral health; ‘moderate oral health’ when the caregiver perceived their child to have ‘neither bad nor good oral health’ (1); and ‘bad oral health’ (2) when they perceived it as, ‘bad’ or ‘very bad’. For the regression analyses, the variable was dichotomised into ‘good oral health’ (0) when the caregiver perceived their child to have ‘good’ or ‘very good’ oral health, and ‘bad oral health’ (1) when they perceived it as, ‘neither bad nor good’, ‘bad’ or ‘very bad’. Similarly, *‘perception of appearance’* was defined as the caregiver being ‘satisfied’ with the appearance of their child’s teeth (0) when the caregiver responded ‘agree’ or ‘strongly agree’; ‘neutral’ when the responses were ‘neither agree nor disagree’ (1); or as ‘dissatisfied’ (2) when the responses were, ‘disagree’ or ‘strongly disagree’. For the regression analyses, the variable was dichotomised into ‘satisfied’ with the appearance of their child’s teeth (0) when the caregiver responded ‘agree’ or ‘strongly agree’, or as ‘dissatisfied’ (1) when the responses were ‘neither agree nor disagree’, ‘disagree’ or ‘strongly disagree’.

#### Caregiver’s awareness

Four items were used to assess the caregiver’s awareness of oral health, which consisted of statements that the respondents were asked to agree (yes) or disagree (no) with: 1) not cleaning teeth can cause tooth decay 2) using fluoridated toothpastes can prevent tooth decay 3) frequent intake of sugared foods and drinks can cause tooth decay and 4) Sugared milk/drinks in a bottle during the night can cause tooth decay.

The variable constructed based on the score for these four items was called *‘caregiver’s caries knowledge’* and was dichotomised into ‘poor knowledge’ (1) for respondents who did not answer any question correctly or who gave only one correct answer, and ‘good knowledge’ (0) for those who answered 2–4 of the questions correctly.

#### Oral hygiene practices (child and caregiver)

The items for oral hygiene practices concerned both the child and the caregiver. Three constructed variables defining the frequency of tooth brushing for the child *‘brushing’* and the caregiver *‘caregiver’s brushing’* and the frequency of using fluoridated toothpaste for the child *‘fluoride’* were categorised as ‘frequent’ (0) when the activities were performed ‘several times a day’ or ‘daily’ and ‘not frequent’ (1) when brushing and fluoridated toothpaste use was ‘seldom or never’.

#### Dental services utilisation (child and caregiver)

The utilisation of dental services by both the child and the caregiver was covered by three constructed variables: *‘child’s dental services utilisation’*, *‘caregiver’s dental services utilisation’* and *‘dentist availability’* nearby, which were categorised into ‘yes’ (0) and ‘no’ (1). The subjective need for dental care was based on items that assessed the caregiver’s reports about their child’s oral complaints during the 6 months preceding the interview (toothache, abscess related to a carious tooth, dry mouth, infected sore gums, bleeding gums, tooth decay or a broken tooth). The constructed variable *‘complaint’* was constructed based on these complaints and dichotomised into ‘yes’ (1) when the caregiver reported that the child had a complaint regarding any of these issues, and ‘no’ (0) when they had no complaints. In addition, a set of items was used to clarify the child’s medical history for the 3 months prior to the study, which covered: 1) antibiotic use; 2) history of hospitalisation; 3) current treatment; and 4) history of medication. The constructed variable *‘child’s medical history’* was dichotomised into ‘yes’ (1) when the caregiver responded affirmatively to any of the four items or ‘no’ (0) when the responses were no in the all medical history items.

### Dependent variables (Outcomes variables)

Two dichotomous dependent variables were used in separate logistic regression analyses: 1) *‘caries’* (DMFT/dmft > 0 or DMFT/dmft = 0); and 2) *‘gingivitis’* (gingival index [GI] > 2 or ≤ 2 [median used as a cut-off]), which were categorised as high (1) or low (0) according to Rios et.al. [30]. Both *‘caries’* and *‘gingivitis’* were significantly more prevalent among CHD cases than controls in our previous study [22] (caries: 66.6 vs 46.7% and gingivitis: 57.3 vs 42.7%, respectively). Additional information on examination methods, diagnostic criteria and findings concerning caries and gingivitis can be found in the previous study [22]. The caries examination was based on the World Health Organization criteria and was recorded by using the dmft/DMFT indices [31]. A simplified form of GI [32] was used to measure gingivitis in the following teeth (55/16, 51/11, 65/26, 75/36, 71/31 and 85/46).

A third variable, *‘child’s dental services utilisation’*, categorised as ‘yes’ (0) if the child had ever used dental services and ‘no’ (1) if they had not, was used as an independent variable to evaluate the relationship with the dependent variables *‘caries’*, and thereafter on *‘gingivitis’*. The variable was also used as a dependent variable in a logistic regression analysis; this time to evaluate the influence of that the various background factors had on the child’s dental services’ utilisation.

#### Anderson’s model

Anderson’s model is a behavioural model which was developed to investigate factors influencing the utilisation of health services [33]. The model proposed that the use of health service is not only influenced by the individual’s need for care (need-related factors), but it’s also the function of several other factors including the sociodemographic (predisposing factors), socioeconomic and other individual-family factors (enabling factors), and the interplay of these factors [33].

**Fig. 1:**
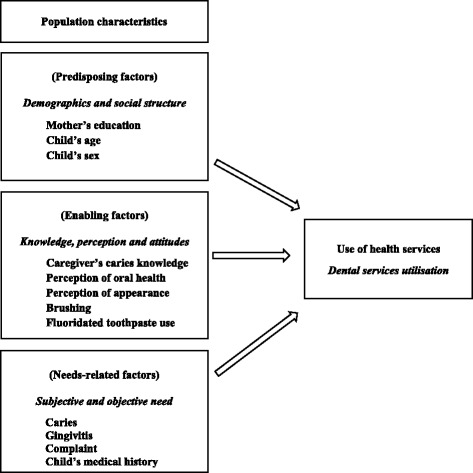
Modified health service use according to Anderson’s model

The dichotomised independent background variables under examination for their association with the dependent variable ‘*child’s dental services utilisation*’ were arranged into categories according to the modified health service use model by Anderson (Fig. 1) [33], which was used in a previous study in adults in Sudan [34]. Variables were categorised into: predisposing factors *(‘mother’s education’, ‘child’s age’* and *‘child’s sex’*); enabling factors (‘*caregiver’s caries knowledge’, ‘perception of oral health’, ‘perception of appearance’, ‘brushing’* and ‘*fluoridated toothpaste use’*); and need-related factors (‘*caries’, ‘gingivitis’, ‘complaint’* and *‘child’s medical history’*).

### Statistical methods

Data were analysed using SPSS version 22 (IBM Corp., Armonk, NY, USA), followed by data cleaning and management. Frequency and percentage tables were created for descriptive statistics for categorical variables. The *χ* ^2^ test was performed to compare statistical differences between CHD cases and controls, except when the expected value in one of the categories occurred in fewer than five children; in which case, Fisher’s exact test was applied. All background variables that differed significantly between CHD cases and controls were included in two separate multiple (adjusted) logistic regression analyses to investigate their effect on the dependent variables (*‘caries’* and *‘gingivitis’*) among CHD cases and then among controls. A further multiple (adjusted) logistic regression analysis was performed to determine the adjusted differences between CHD cases and controls *‘status’* in the dependent variables (*‘caries’* and *‘gingivitis’*) while controlling for the effect of background factors. Also, a hierarchical logistic regression model was developed to examine the effect of predisposing, enabling and need-related factors on the third dependent variable *‘child’s dental services utilisation’* in a separate regression analysis. In the model, Step 1 controlled for predisposing factors and then enabling and need-related factors were entered in Steps 2 and 3, respectively. Odds ratios (ORs) with 95% confidence intervals (CIs) were used to estimate the association. *p* < 0.05 were considered statistically significant. Nagelkerke R^2^ values were presented to indicate the amount of variation in the dependent variable explained by the model.

## Results

The response rates for CHD cases and controls were 95.7 and 94.8%, respectively. The mean ages of CHD cases and controls were 7.1 years (standard deviation [SD 3]) and 7.2 years (2.8), respectively.

### Frequency distribution of oral-health-related background variables among CHD cases and controls

Table 1 shows that the frequency distribution of oral-health-related background variables differed significantly between CHD cases and controls in both age groups. In age group 1, a significantly lower percentage of caregivers perceived that their child had good oral health among CHD cases than among controls. In both age groups, good knowledge of caries among caregivers and frequent brushing and use of fluoridated toothpaste by the child were significantly less common among CHD cases than among controls.

**Table 1:** Frequency distribution of oral- health-related background variables in age groups 1 and 2

	Age group 1 (3–7 years)			Age group 2 (8–12 years)		
	CHD cases	Controls	*p*- value	CHD cases	Controls	*p*- value
Caregiver’s perceptions about their child’s dental conditions						
Perception of oral Health						
Good	35 (57.4%)	76 (76%)	0.027*	31 (63.3%)	47 (59.9%)	ns
Moderate	9 (14.8%)	5 (5%)		7 (14.3%)	7 (8.9%)	
Bad	17 (27.9%)	19 (19%)		11 (22.4%)	25 (31.6%)	
Perception of appearance						
Satisfied	46 (75.4%)	77 (77%)	ns	31 (63.3%)	51 (64.6%)	ns
Neutral	6 (9.8%)	3 (3%)		5 (10.2%)	6 (7.6%)	
Unsatisfied	9 (14.8%)	20 (20%)		13 (26.5%)	22 (27.8%)	
Caregiver’s awareness (caregiver’s caries knowledge)						
Good knowledge	52 (85.2%)	97 (97%)	0.010*	45 (93.8%)	75 (100%)	0.001**
Oral hygiene practices						
Brushing						
Frequent	46 (74.2%)	93 (93%)	0.002**	42 (85.7%)	78 (98.7%)	0.003**
Fluoride						
Frequent	27 (43.5%)	72 (72%)	0.001**	27 (55.1%)	61 (77.2%)	0.011*
Caregiver’s brushing						
Frequent	62 (100%)	100 (100%)	ns	49 (100%)	76 (97.4%)	ns
Utilisation of dental services						
Child’s dental services utilisation						
Yes	5 (8.1%)	11 (11%)	ns	19 (38.8%)	28 (34.6%)	ns
Dentist availability						
Yes	41 (66.1%)	89 (89%)	0.001**	32 (65.3%)	62 (81.6%)	0.040*
Caregiver’s dental services utilisation						
Yes	20 (32.3%)	37 (37%)	ns	14 (28.6%)	46 (59.0%)	0.001**

Age group 1 (CHD cases: *n* = 62; controls: *n* = 101) and age group 2 (CHD cases: *n* = 49; controls: *n* = 81). The χ2test was used for the comparisons. (ns) denotes not significant, *denotes significance at *p* < 0.05 and **significance at *p* < 0.01

Regarding dental services utilisation, caregivers of CHD cases reported significantly less availability of a nearby dentist compared with the caregivers of controls in both age groups. In age group 2, a significantly higher percentage of the caregivers of controls reported attending the dentist compared with the caregivers of CHD cases. Regarding the ‘*complaint*’ variable, a significantly higher percentage of CHD cases compared with controls in age group 1 reported having a dry mouth (25.8% CHD cases vs 4% controls) and infected gums (14.8% CHD cases vs 5% controls). Regarding the child’s medical history, 19% of CHD cases versus 2.7% of controls reported a history of hospitalisation, whereas 46.8% of CHD cases versus 45.6% of controls reported antibiotic use during the 3 months preceding the study. Also, about 20.7% of CHD cases were chronic users of medication and 30.6% were currently receiving treatment; both of these items were related to their cardiac defects.

### Impact of independent variables on dependent variables ‘caries’ and ‘gingivitis’ within CHD cases and controls and between CHD cases and controls

The effects of background factors (independent variables) on the dependent variables ‘*caries*’ and ‘*gingivitis*’ (within CHD cases and within controls) are shown in Tables 2 and 3, respectively. In CHD cases, the ‘*brushing*’ and ‘*fluoride*’ variables had a significant effect on the prevalence of caries, with ORs of 5.6 (95% CI: 1.4–22.8) and 0.3 (95% CI: 0.13–0.8), respectively, among infrequent brushers and fluoride toothpaste compared with frequent users, as well as the mother’s level of education (OR = 2.6, 95% CI: 1.0–6.4). In controls, the variable ‘*mother’s education*’ had a significant effect on the dependent variable ‘*gingivitis*’, with an OR of 2.2 (95% CI: 1.1–4.6) for those with an uneducated mother compared with those with an educated mother.

**Table 2:** Multiple logistic regression analysis of oral- health-related background factors (independent variables) against caries (dependent variable) within cases and controls

	CHD cases (*n* = 111)	Controls (*n* = 182)
Independent variables	Multiple analysis Adjusted OR (95% CI)	Multiple analysis Adjusted OR (95% CI)
Brushing		
Frequent (R)	1	1
Not frequent	5.60 (1.38–22.81)**	4.09 (0.72–23.32)
Fluoride		
Frequent (R)	1	1
Not frequent	0.32 (0.13–0.84)*	0.98 (0.46–2.09)
Caregiver’s caries knowledge		
Good knowledge (R)	1	1
Low knowledge	3.082.05 (0.38–11.06)	2.78 (0.25–31.79)
Mother’s education		
Higher education (R)	1	1
Lower education	2.59 (1.04–6.44)**	1.04 (0.51–2.10)
Child’s dental services utilisation		
Yes (R)	1	1
No	1.82 (0.65–5.10)	0.66 (0.32–1.37)

Oral-health-related background factors (independent variables) were regressed against the dependent variable (Decayed, missed and filled teeth; DMFT/dmft > 0 and DMFT/dmft = 0). Multivariate analyses were performed using logistic regression, where R denotes the reference for analysis with odd ratio OR = 1. *denotes significance at *p* < 0.05 and **significance at *p* < 0.01

**Table 3:** Multiple logistic regression analysis of oral- health-related background factors (independent variables) against gingivitis (dependent variable) within cases and controls

	CHD cases (*n* = 111)	Controls (*n* = 182)
Independent variables	Multiple analysis Adjusted OR (95 % CI)	Multiple analysis Adjusted OR (95 % CI)
Brushing		
Frequent (R)	1	1
Not frequent	1.90 (0.54–6.75)	1.08 (0.18–6.49)
Fluoride		
Frequent (R)	1	1
Not frequent	1.07 (0.42–2.72)	0.56 (0.24–1.32)
Caregiver’s caries knowledge		
Good knowledge (R)	1	1
Low knowledge	1.16 (0.28–4.86)	0.87 (0.07–10.43)
Mother’s education		
Higher education (R)	1	1
Lower education	1.22 (0.49–3.02)	2.21 (1.06–4.60)**
Child’s dental services utilisation		
Yes (R)	1	1
No	0.28 (0.08–1.07)	0.86 (0.39–1.87)

Oral-health-related background factors (independent variables) were regressed against the dependent variable (gingivitis: gingival index GI > 2 and GI ≤ 2). Multivariate analyses were performed using logistic regression, where R denotes the reference for analysis with odd ratios OR = 1. **donates﻿ significance at *p* < 0.01

No significant interaction effects between background factors and having a CHD were evident for the dependent variables ‘*caries*’ and ‘*gingivitis*’ (Additional file 1: Tables S1 and Additional file 2: Table S2). Tables 4 and 5 show the adjusted differences in the prevalence of caries and gingivitis between the CHD cases and controls while controlling for background factors (adjusted ORs). The adjusted ORs for ‘*caries*’ and ‘*gingivitis*’ in CHD cases compared with controls were 1.8 (95% CI: 1.1–3.2) and 5.3 (95% CI: 2.9–9.4), respectively, when all other background factors were controlled for.

**Table 4:** Adjustment for effect of oral-health-related background factors on difference in dependent variable ‘caries’ between cases and controls

	Crude analysis Unadjusted OR (95% CI)	Multiple analysis Adjusted OR (95% CI)
Status		
Controls (R)	1	1
CHD cases	2.28 (1.39–3.73) **	1.84 (1.07–3.16) **
Brushing		
Frequent (R)	1	1
Not frequent	5.08 (1.89–13.64)**	5.02 (1.71–14.74)**
Fluoride		
Frequent (R)	1	1
Not frequent	1.15 (0.71–1.86)	0.63 (0.35–1.22)
Caregiver’s caries knowledge		
Good knowledge (R)	1	1
Low knowledge	3.68 (1.02–13.36)**	2.46 (0.64–9.47)
Mother’s education		
Higher education (R)	1	1
Lower education	1.46 (0.69–3.11)	0.97 (0.88–2.61)
Child’s dental services utilisation		
Yes (R)	1	1
No	1.01 (0.58–1.77)	0.91 (0.51–1.65)

Logistic regression analysis (bivariate and multivariate) was used to determine whether there was a difference in the prevalence of caries (Decayed, missed and filled teeth; DMFT/dmft > 0 and DMFT/dmft = 0) between CHD cases (*n* = 111) and controls (*n* = 182) while controlling for the different oral- health-related background variables (independent variables). Odds ratios (ORs) are presented with 95% confidence intervals (CIs). **donates﻿ significance at *p* < 0.01

**Table 5:** Adjustment for effect of oral-health-related background factors on difference in dependent variable ‘gingivitis’ between cases and controls

	Crude analysis Unadjusted OR (95% CI)	Multiple analysis Adjusted OR (95% CI)
Status		
Controls (R)	1	1
CHD cases	5.61 (3.32–9.47)**	5.29 (2.98–9.39)**
Brushing		
Frequent (R)	1	1
Not frequent	2.39 (1.09–5.29)**	1.51 (0.58–3.97)
Fluoride		
Frequent (R)	1	1
Not frequent	1.39 (0.86–2.27)	0.72 (0.39–1.33)
Caregiver’s caries knowledge		
Good knowledge (R)	1	1
Low knowledge	2.24 (0.74–6.72)	0.98 (0.29–3.38)
Mother’s education		
Higher education (R)	1	1
Lower education	4.34 (1.81–10.38)**	1.74 (0.99–3.07)*
Child’s dental services utilisation		
Yes (R)	1	1
No	0.77 (0.44–1.36)	1.58 (0.84–2.95)

Logistic regression analyses (bivariate and multivariate) were used to ascertain the exact difference in the prevalence of gingivitis (gingival index GI > 2 and GI ≤ 2) between CHD cases (*n* = 111) and controls (*n* = 182) while controlling for the different oral- health-related background variables (independent variables). Odds ratios (ORs) are presented with 95% confidence intervals (CIs). *denotes significance at *p* < 0.05 and **significance at *p* < 0.01

### Impact of independent variables on dependent variable ‘child’s dental services utilisations’

The effects of background factors on the child’s use of dental services (‘*child’s dental services utilisation*’), based on hierarchical logistic regression analyses are presented in Table 6. This shows that the predisposing factors in Step 1 explained 19.4% (Nagelkerke’s R^2^, *p* < 0.05) of the variation in the child’s dental services utilisation (in the total sample). This increased to 27.5% (Nagelkerke’s R^2^, *p* < 0.01) in Step 2, with the addition of enabling factors and to 33.4% (Nagelkerke’s R^2^, *p* < 0.01) when need-related factors were added in Step 3. The stratified analyses showed that the model explained 49.7% of the variation in the dental services utilisation among CHD cases, whereas it explained 34.6% of the variation among controls.

**Table 6:** Child’s dental services utilisation regressed on predisposing, enabling and need-related factors

	Total sample	CHD cases (111)	Controls (182)
Step 1: Predisposing factors	Adjusted OR (95% CI)	Adjusted OR (95% CI)	Adjusted OR (95% CI)
Mother’s education			
Higher education (R)	1	1	1
Lower education	0.36 (0.16–0.80)*	0.16 (0.03–0.89)*	0.62 (0.211.85)
Child’s age			
Age 1 (R)	1	1	1
Age 2	5.26 (2.40–11.52)**	11.89 (1.98– 71.56)**	5.17 (1.86– 14.38)**
Child’s sex			
Boy (R)	1	1	1
Girl	1.46 (0.74–2.89)	1.78 (0.45–7.02)	1.46 (0.61– 3.46)
Nagelkerke’s R^2^	0.194	0.248	0.169
Step 2: Enabling factors			
Caregiver’s caries knowledge			
Good knowledge (R)	1	1	1
Low knowledge	0.48 (0.06–4.27)	0.88 (0.07–10.51)	-
Perception of oral health			
Good (R)	1	1	1
Bad	1.32 (0.61–2.84)	3.82 (0.89–16.39)	0.69 (0.25–1.91)
Perception of appearance			
Satisfied	1	1	1
Unsatisfied	1.89 (0.92–3.92)	1.04 (0.28–3.88)	2.95 (1.14–7.59)*
Brushing			
Frequent (R)	1	1	1
Not frequent	0.88 (0.24–3.33)	1.05 (0.19–5.77)	0.76 (0.06–10.12)
Fluoride			
Frequent (R)	1	1	1
Not frequent	0.85 (0.39–1.88)	0.61 (0.15–2.48)	0.97 (0.32–2.95)
Nagelkerke’s R^2^	0.275	0.421	0.231
Step 3: Need-related factors			
Caries			
DMFT = 0 (R)	1	1	1
DMFT > 0	1.15 (0.53–2.49)	0.51 (0.12–2.37)	1.83 (0.68–4.91)
Gingivitis			
GI > 2 (R)	1	1	1
GI ≤ 2	0.86 (0.41–1.80)	8.23 (0.52–28.60)	0.39 (0.15–1.05)
Complaint			
No (R)	1	1	1
Yes	4.41 (1.78–10.87)**	6.71 (0.99–45.25)	6.36 (1.96–20.63)**
Child’s past medical history			
No (R)	1	1	1
Yes	1.33 (0.68–2.61)	2.13 (0.54–8.45)	1.14 (0.48–2.70)
Nagelkerke’s R^2^	0.334	0.497	0.346

Hierarchical logistic regression analyses for effect of oral-health-related background variables categorised according to Anderson’s model into predisposing, enabling and need-related factors. Analyses were performed on the total for CHD cases and controls. Odds ratios (ORs) are presented with 95% confidence intervals (Cis). *denotes significance at *p* < 0.05 and **significance at *p* < 0.01

The factors that significantly affected the child’s dental services utilisation among CHD cases were: 1) the child’s age, with an OR of 11.9 (95% CI: 1.9–71.6) for children in age group 2 compared with children in age group 1; and 2) mother’s level of education with an OR of 0.16 (95% 0.03–0.89) for children with lower educated mothers compared with children with higher education mothers.

Regarding the controls, the child’s age (age group 2: OR = 5.2, 95% CI: 1.9–3.5) had a significant effect on the child’s dental visits as well as the caregiver’s perceptions of their child’s dental appearance, with an OR of 2.9 (95% CI: 1.1–7.6) for caregivers who were unsatisfied with their child’s dental appearance compared with caregivers who were satisfied with their child’s oral health. The ‘*complaint*’ variable also had a significant impact on the dental services utilisation among controls, with an OR of 6.4 (95% CI: 1.9–20.6) for children with complaints compared with those without complaints.

## Discussion

This study investigated the oral-health-background factors and dental services utilisation among Sudanese children with CHDs. These children constitute a high-risk group with high susceptibility to oral diseases that may directly influence their general health, therefore they warrant investigation. The study was undertaken to provide the authorities with important information and highlight areas of insufficiency for future preventive oral health programmes that might reduce the injurious consequences of oral disease among this group. The current study revealed that children with a CHD exhibited a significantly higher percentage of dental risk predictors for both caries and gingivitis than their healthy peers did. More than 20% of the caregivers of CHD cases reported that their children did not brush their teeth frequently, which was significantly higher than the figure recorded for the controls. The constructed variable ‘*brushing*’ in the logistic regression analyses showed a predominant significant association with the dependent variable ‘*caries*’ among CHD cases, with an OR of >5 among CHD cases, in accordance with an earlier UK-based study [35]. This indicates that a child with CHD who is an infrequent brusher has a risk of caries five times higher than a child with CHD who is a frequent brusher. Also, two UK-based and one Indian study among children with a CHD have reported similar low brushing frequencies that were considered unsatisfactory [3, 13, 14]. Additionally, caregivers of CHD cases achieved significantly lower caries knowledge scores compared with the caregivers of the controls. Those findings indicated a clear lack of adequate oral hygiene measures among the CHD cases as well as lack of sufficient oral health knowledge among their caregivers.

The previously reported higher prevalence of caries and gingivitis among children with a CHD compared with those without a CHD in our previous study [22] has been strengthened by the results of the present logistic regression analyses. The CHD cases had a higher likelihood (ORs) of caries and gingivitis compared with the controls, even after controlling for the effects of differences in background factors. Notably, this could merit further investigation of other background factors not examined in the current study that might have an impact on those children’s oral health. Hierarchical logistic regression analyses showed that the child’s age and mother’s level of education were significantly associated with the dental services utilisation among CHD cases. These findings also indicate that it is essential to encourage caregivers of children with a CHD to utilise dental services. Beyond doubt, these findings will be of use for any targeted future preventive oral health care programmes for children with a CHD in the studied population. There are reasons to believe that the findings are representative of the Khartoum State as a whole. Participation response rates were excellent; the controls were well adjusted to the CHD cases because they were selected from several schools and kindergartens in Khartoum State using stratified random sampling and were matched to CHD cases by group matching [22].

More than 40% of the caregivers of CHD cases were aware of the deterioration of their child’s dental health (constructed variable ‘*perception of oral health*’), yet they had not taken their child to a dentist. Indeed, in our previous study [22], two children with a CHD exhibited swelling and sinus discharge related to carious teeth at the time of data collection and had never been taken to a dentist. In addition, the caregivers of CHD cases were less knowledgeable about caries, but this might indicate a lack of proper dental health information. In Sweden and the UK, as a comparison, the caregivers of children with a CHD who are registered with a dental practitioner have significantly better dental knowledge than the caregivers of children who are not registered [17]. Also, children with CHD and their caregivers are given comprehensive dental care and participate in a robust programme on the prevention of caries [16]. Nevertheless, most interventions for caries in the Swedish children took place after caries progression, because continuous hospital admissions delayed their ability to attend a dentist for treatment [16]*.*

In terms of children’s complaints, the percentages of those with dry mouth and infected sore gums were higher among CHD cases than controls. This may be explained by the use of medication related to underlying cardiac problems in the CHD cases; some of which are known to cause xerostomia [35, 36] and, consequently, sore gums.

The mother’s level of education is known to be an important predictor of children’s oral health [37, 38]. In this study, this variable was found to have a significant effect on the prevalence of caries among CHD cases and gingivitis among controls.

An unexpected finding was the effect that use of fluoridated toothpaste had on the prevalence of caries among CHD cases. Children with a CHD who frequently used fluoridated toothpaste had a significantly higher likelihood of caries than infrequent users. One explanation may be that the amount of fluoride in the toothpaste and frequency of brushing were insufficient to confer full protection. Also, the CHD cases represent a group in need of extra means of fluoride application. Another possible explanation may be that the contribution of other background factors to the deterioration of the oral health of children with a CHD might have reduced the effectiveness of fluoride against dental caries.

About 20% of the caregivers of CHD cases reported that they had visited a dentist to seek dental treatment for their child’s complaint, but never for preventive check-ups, whereas the remaining 80% stated that they had never visited the dentist with their child for regular preventive check-ups or for dental treatment. Thus, the percentage of those who had never visited a dentist was much higher compared with that in three previous UK-based studies on dental visits among children with a CHD (18, 9 and 19% of had never visited a dentist, respectively) [3, 12, 14] and a recent Brazilian study (29% had never visited a dentist) [10].

A significantly higher percentage of caregivers of CHD cases in both age groups reported that they had no available nearby dental care, in significantly higher percentages compared with controls. CHD cases and controls were considered to live in similar surroundings; therefore, the higher proportion of caregivers reporting the unavailability of nearby dental care was challenging to interpret. One possible explanation is that the burden on the caregiver of the health condition of their child may have had a negative effect on their awareness of the availability of nearby dental care. Another possible explanation is that, in general, Sudanese people seldom seek dental services because of lack of public funding and insurance coverage for most dental treatment and the high cost of the treatment available [25, 34]. In addition, many children with a CHD are more fearful of medical staff, so the idea of dental treatment itself, together with a lack of dental staff knowledgeable in handling such CHD cases, may also have had an impact on attendance at dental clinics [35].

Regarding the dental services utilisation, Anderson’s model is one of the extensively used models that investigated factors influencing families’ and individuals’ utilisation of the health services [39]. This model was developed by Anderson during the 60s and went through several developments since then [33]. The model had also been applied in the field of dentistry [40, 41] and had been recently used among Sudanese adults in Khartoum State, Sudan [34]. Therefore, the same model was chosen for the categorisation of the variables in the current hierarchical logistic analysis. Hierarchical logistic regression analysis showed that the mother’s level of education, and the child’s age were the most significant factors in explaining the child’s dental services utilisation among both CHD cases and controls. This regression analysis also highlighted that, among CHD cases, a higher level of mother’s education and older age of the child equated to a higher number of dental visits. The influence of age may be explained by a tendency to focus more on the child’s permanent dentition than on primary teeth when seeking dental care.

## Conclusions

The patterns of the oral-health-background factors differed significantly between CHD cases and controls with lower frequencies of brushing and fluoridated toothpaste use among CHD cases as well as their caregivers’ lower scores regarding knowledge of caries. The effects of ‘brushing’ and ‘mother’s level of education’ were significantly influencing caries prevalence among CHD cases and those children still had higher likelihood for both caries and gingivitis compared with controls even after controlling for their differences in the background factors. Finally, the influences of ‘child’s age’ and ‘mothers’ level of education’ in the child’s utilisation of dental services were significant among CHD cases with higher reported dental visits among those aged 8–12 years and those having mothers with higher education.

The study findings indicate that there is a need in Sudan for preventive oral health programmes for children with a CHD, focusing on oral hygiene education as well as regular preventive dental visits.

## Additional files

Additional file 1: Table S1. Interaction effects of oral-health-related background factors and having a CHD on prevalence of caries (DMFT/dmft > 0 and DMFT/dmft = 0). Interaction effects of oral-health-related background variables on prevalence of caries were ascertained using bivariate logistic regression analysis of the variables among CHD cases and controls. Odds ratios (ORs) are presented with 95% confidence intervals (CIs). (ns) denotes not significant, *denotes significance at *p* < 0.05 and **significance at *p* < 0.01. (DOCX 15 kb)
Additional file 2: Table S2. Interaction effects of oral-health-related background factors and having a CHD on the prevalence of gingivitis (gingival index GI >2 and ≤2). Interaction effects of oral- health-related background variables on prevalence of gingivitis were ascertained using bivariate logistic regression analysis of the variables among CHD cases and controls. Odds ratios (ORs) are presented with 95% confidence intervals (CIs). (ns) denotes not significant, *denotes significance at *p* < 0.05 and **significance at *p* < 0.01. (DOCX 15 kb)

## Abbreviations

CHDs: Congenital heart defects
CI: Confidence interval
dmft/DMFT: Decayed missed and filled teeth
GI: Gingival index
OR: Odd ratio
SD: Standard deviation
