# Supplementary material for: Oral-health-related background factors and dental service utilisation among Sudanese children with and without a congenital heart defects
Source: BMC Oral Health. 2016 Nov 15;16:123. doi: 10.1186/s12903-016-0318-5 (PMC5111257; doi:10.1186/s12903-016-0318-5)
Supplement: Additional file 1: Table S1. — Interaction effects of oral-health-related background factors and having a CHD on prevalence of caries (DMFT/dmft > 0 and DMFT/dmft = 0). Interaction effects of oral-health-related background variables on prevalence of caries were ascertained using bivariate logistic regression analysis of the variables among CHD cases and controls. Odds ratios (ORs) are presented with 95% confidence intervals (CIs). (ns) denotes not significant, *denotes significance at p < 0.05 and **significance at p < 0.01. (DOCX 15 kb) [file 12903_2016_318_MOESM1_ESM.docx]

**Table S1. Interaction effects of oral- health-related background factors and having a CHD on prevalence of caries (DMFT/dmft > 0 and DMFT/dmft = 0)**

|  | **CHD cases (111)** | **Controls (182)** | **Interactions** |
| --- | --- | --- | --- |
|  |  |  |  |
| **Independent**  **variable** | **Crude analysis**  **Unadjusted OR (95% CI)** | **Crude analysis**  **Unadjusted OR (95% CI)** |  |
| **Brushing**  Frequent (R)  Not frequent | **4.19 (1.16-15.21)**** | 3.66 (0.72-18.66) | 1.15 (0.14-9.14) |
| **Fluoride**  Frequent (R)  Not frequent | 0.72 (0.33-1.59) | 1.08 (0.55-2.11) | 0.67 (0.24-1.88) |
| **Caregiver’s caries knowledge**  Good knowledge (R)  Low knowledge | 2.82 (0.59-13.62) | 2.41 (0.22-27.08) | 1.17 (0.07-20.98) |
| **Mother’s education**  Higher education (R)  Lower education | 2.87 (1.26-6.51)** | 0.96 (0.49-1.85) | 1.13 (0.38-3.36) |
| **Child’s dental services utilisation**  Yes (R)  No | 1.99 (0.79-5.00) | 0.68 (0.33-1.39) | 2.91 (0.91-9.36) |

Interaction effects of oral- health-related background variables on prevalence of caries were ascertained using bivariate logistic regression analysis of the variables among CHD cases and controls. Odds ratios (ORs) are presented with 95% confidence intervals (CIs). (ns) denotes not significant, * denotes significance at *p* < 0.05 and **significance at *p* < 0.01.
