# Supplementary material for: Oral-health-related background factors and dental service utilisation among Sudanese children with and without a congenital heart defects
Source: BMC Oral Health. 2016 Nov 15;16:123. doi: 10.1186/s12903-016-0318-5 (PMC5111257; doi:10.1186/s12903-016-0318-5)
Supplement: Additional file 2: Table S2. — Interaction effects of oral-health-related background factors and having a CHD on the prevalence of gingivitis (gingival index GI >2 and ≤2). Interaction effects of oral- health-related background variables on prevalence of gingivitis were ascertained using bivariate logistic regression analysis of the variables among CHD cases and controls. Odds ratios (ORs) are presented with 95% confidence intervals (CIs). (ns) denotes not significant, *denotes significance at p < 0.05 and **significance at p < 0.01. (DOCX 15 kb) [file 12903_2016_318_MOESM2_ESM.docx]

**Table S2. Interaction effects of oral-** **health-related background factors and having a CHD on the prevalence of gingivitis (gingival index: GI >2** **and ≤2).**

|  | **CHD cases (111)** **Controls (182)** | | **Interactions** |
| --- | --- | --- | --- |
| **Independent variables** | **Crude analysis**  **Unadjusted OR (95% CI)** | **Crude analysis**  **Unadjusted OR (95% CI)** |  |
| **Brushing**  Frequent (R)  Not frequent | 1.89 (0.58-6.09) | 0.65 (0.13-3.32) | 2.90 (0.39-21.65) |
| **Fluoride**  Frequent (R)  Not frequent | 1.18 (0.51-2.76) | 0.72 (0.35-1.50) | 1.64 (0.53-5.03) |
| **Caregiver’s caries knowledge**  Good knowledge (R)  Low knowledge | 1.09 (0.28-4.37) | 1.04 (0.09-11.66) | 1.06 (0.07-17.24) |
| **Mother’s education**  Higher education (R)  Lower education | 1.24 (0.53-2.89) | 1.86 (0.94-3.67) | 1.36 (0.39-4.66) |
| **Child’s dental services utilisation**  Yes (R)  No | 0.34 (0.09-1.22) | 1.01 (0.48-2.15) | 0.33 (0.07-1.48) |

Interaction effects of oral- health-related background variables on prevalence of gingivitis were ascertained using bivariate logistic regression analysis of the variables among CHD cases and controls. Odds ratios (ORs) are presented with 95% confidence intervals (CIs). (ns) denotes not significant, * denotes significance at *p* < 0.05 and ** significance at *p* < 0.01.
